# Supplementary material for: Intranasal or airborne transmission-mediated delivery of an attenuated SARS-CoV-2 protects Syrian hamsters against new variants
Source: Nat Commun. 2023 Jun 9;14:3393. doi: 10.1038/s41467-023-39090-4 (PMC10250859; doi:10.1038/s41467-023-39090-4)
Supplement: Supplementary file 1 — Supplementary Materials [file 41467_2023_39090_MOESM1_ESM.pdf]

Intranasal or airborne transmission-mediated delivery of an attenuated SARS-CoV-2 protects Syrian hamsters against new variants

Authors: Charles B. Stauft[1,†], Prabhuanand Selvaraj[1,†], Felice D'Agnillo[2,†], Clement A. Meseda[1,†], Shufeng Liu[1], Cyntia L. Pedro[1], Kotou Sangare[1], Christopher Z. Lien[1], Jerry P. Weir[1], Matthew F. Starost[3], Tony T. Wang[1]*

Affiliations:

[1]Division of Viral Products, Center for Biologics Evaluation and Research, Food and Drug Administration, Silver Spring, Maryland, USA.

[2]Laboratory of Biochemistry and Vascular Biology, Center for Biologics Evaluation and Research, Food and Drug Administration, Silver Spring, MD, USA.

[3]Division of Veterinary Resources, Diagnostic and Research Services Branch, National Institutes of Health, Rockville Pike, USA.

*Email: Tony.Wang@fda.hhs.gov

Supplementary Materials

Supplementary figures

Fig. S1

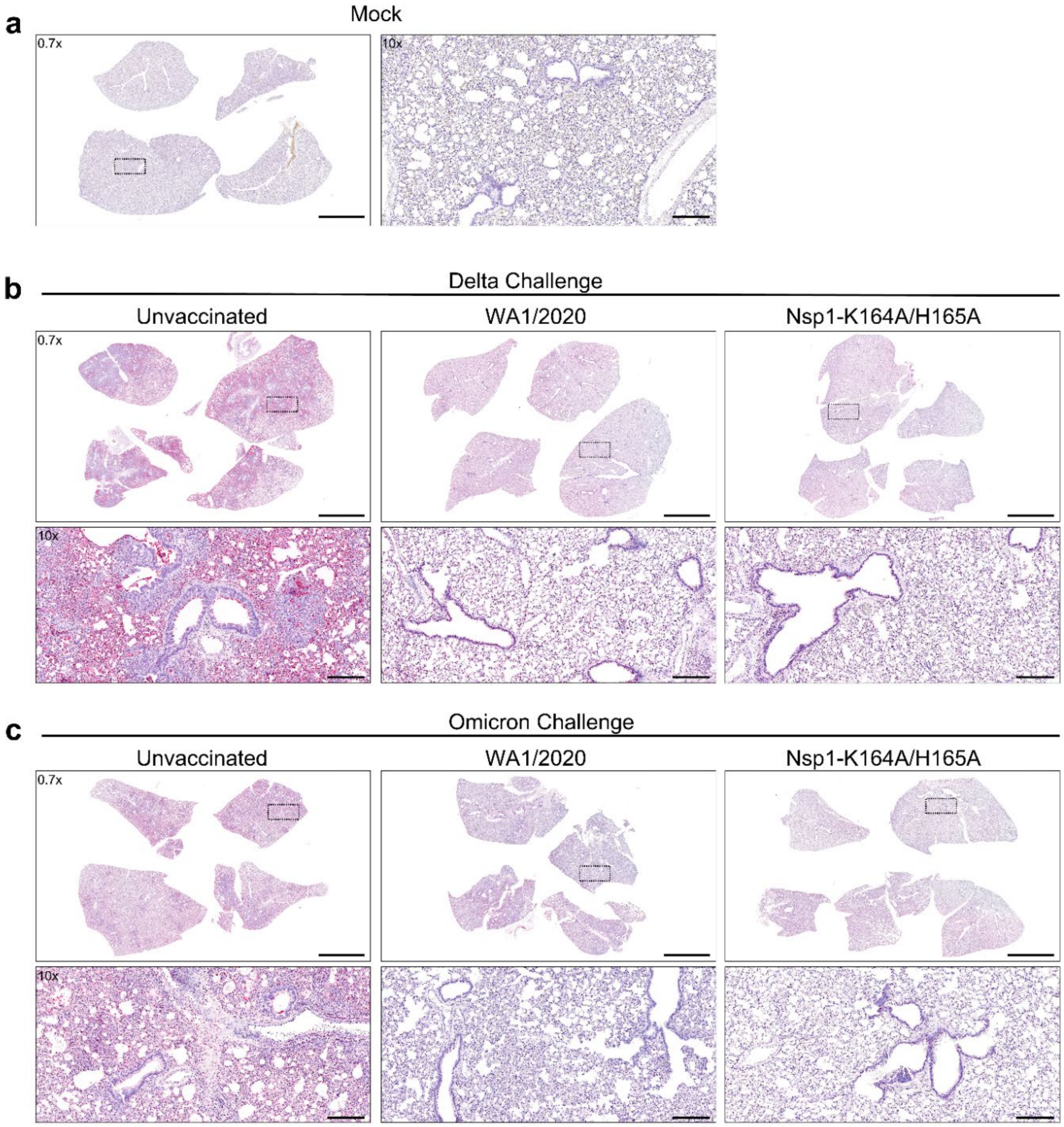

Fig. S1: Nsp1-K164A/H165A protects against TUNEL-positive cell death in hamster lungs post-challenge with Delta and Omicron isolates

Syrian hamsters were vaccinated with Nsp1-K164A/H165A or WA1/2020 35 days prior to challenge with Delta or BA.1 Omicron isolates on day 0. TUNEL reactivity (magenta staining) was examined in lung sections from (**a**) non-infected non-vaccinated hamsters (mock) or

following challenge with (**b**) Delta or (**c**) Omicron at 7 dpi (n = 3 hamsters per group). Nuclei were counterstained with hematoxylin. Black boxes indicate the regions of magnification. Scale bars: 5 mm (0.7x), 250 mm (10x).
