# Supplementary material for: Intranasal or airborne transmission-mediated delivery of an attenuated SARS-CoV-2 protects Syrian hamsters against new variants
Source: Nat Commun. 2023 Jun 9;14:3393. doi: 10.1038/s41467-023-39090-4 (PMC10250859; doi:10.1038/s41467-023-39090-4)
Supplement: Supplementary file 2 — Reporting Summary [file 41467_2023_39090_MOESM2_ESM.pdf]

nature portfolio

Corresponding author(s): Tony T. Wang

Last updated by author(s): Apr 7, 2023

Reporting Summary

Nature Portfolio wishes to improve the reproducibility of the work that we publish. This form provides structure for consistency and transparency in reporting. For further information on Nature Portfolio policies, see our Editorial Policies and the Editorial Policy Checklist.

Statistics

For all statistical analyses, confirm that the following items are present in the figure legend, table legend, main text, or Methods section.

n/a	Confirmed	
☐	☒	The exact sample size (*n*) for each experimental group/condition, given as a discrete number and unit of measurement
☐	☒	A statement on whether measurements were taken from distinct samples or whether the same sample was measured repeatedly
☐	☒	The statistical test(s) used AND whether they are one- or two-sided<br>*Only common tests should be described solely by name; describe more complex techniques in the Methods section.*
☒	☐	A description of all covariates tested
☒	☐	A description of any assumptions or corrections, such as tests of normality and adjustment for multiple comparisons
☐	☒	A full description of the statistical parameters including central tendency (e.g. means) or other basic estimates (e.g. regression coefficient) AND variation (e.g. standard deviation) or associated estimates of uncertainty (e.g. confidence intervals)
☒	☐	For null hypothesis testing, the test statistic (e.g. *F*, *t*, *r*) with confidence intervals, effect sizes, degrees of freedom and *P* value noted<br>*Give P values as exact values whenever suitable.*
☒	☐	For Bayesian analysis, information on the choice of priors and Markov chain Monte Carlo settings
☒	☐	For hierarchical and complex designs, identification of the appropriate level for tests and full reporting of outcomes
☒	☐	Estimates of effect sizes (e.g. Cohen's *d*, Pearson's *r*), indicating how they were calculated

Our web collection on statistics for biologists contains articles on many of the points above.

Software and code

Policy information about availability of computer code

Data collection	Data were collected using Microsoft Excel (Version 2102 , Microsoft Office 365), Agilent Cytation 7 with built-in software (Gen5 version 3.11), Glomax luminometer (Progema), StepOne Plus thermal cycler (Qiagen) with built-in software (Step One (tm) Software version 2.3), NovaSeq 6000 sequencer (Illumina, San Diego, CA), Softmax Pro 7 software (Molecular Devices), Aperio ImageScope (12.4.3.5008), ZEN 2 (version 2.0) imaging software, and NDP.view2 (version 2.7.52) software.
Data analysis	Data were analyzed using Microsoft Excel (Version 2102, Microsoft Office 365), and Graphpad Prism (Version 9.40).

For manuscripts utilizing custom algorithms or software that are central to the research but not yet described in published literature, software must be made available to editors and reviewers. We strongly encourage code deposition in a community repository (e.g. GitHub). See the Nature Portfolio guidelines for submitting code & software for further information.

Data

Policy information about availability of data

All manuscripts must include a data availability statement. This statement should provide the following information, where applicable:

- Accession codes, unique identifiers, or web links for publicly available datasets
- A description of any restrictions on data availability
- For clinical datasets or third party data, please ensure that the statement adheres to our policy

- no accession codes, unique identifiers, or web links for publicly available datasets were included.

-all data are available from the corresponding author
-no clinical datasets or third party data were included.

Human research participants

Policy information about studies involving human research participants and Sex and Gender in Research.

Reporting on sex and gender	none
Population characteristics	none
Recruitment	none
Ethics oversight	n/a

Note that full information on the approval of the study protocol must also be provided in the manuscript.

Field-specific reporting

Please select the one below that is the best fit for your research. If you are not sure, read the appropriate sections before making your selection.

☒ Life sciences ☐ Behavioural & social sciences ☐ Ecological, evolutionary & environmental sciences

For a reference copy of the document with all sections, see nature.com/documents/nr-reporting-summary-flat.pdf

Life sciences study design

All studies must disclose on these points even when the disclosure is negative.

Sample size	No sample size calculation was performed. Sample sizes were chosen based on available literature for animal vaccine studies and our experience with the hamster model of SARS-CoV-2, sample sizes used in this study provide power to determine efficacy differences between vaccinated and non-vaccinated control groups. For hamster studies, n≥3 was chosen for each experimental group for calculating statistical significance in the study. In most experiments, however, there are at least 4 hamsters per group. For the study involving mice, n≥5 was chosen for each experimental group. For in vitro experiments, there are 2-6 technical replicates for each treatment/group. These sample sizes are chosen in conformity with similar studies in the field published by Nature journals in the last year (Liu et al, 2022; Nouailles et al, 2023; Wussow et al, 2023; Sharma et al, 2022).
Data exclusions	No data were excluded from the study.
Replication	In vitro experiments were repeated twice. Results from animal studies were obtained either from one independent experiment or pooled from two separate experiments with age-matched animals. All attempts at replication were successful.
Randomization	Animals in this study were randomized into different groups for experiments.
Blinding	Investigators were not blinded in data acquisition or sample collection because it was not possible to conduct the BSL-3 experiment without knowing such information. However, after sample collection, only a sample ID was available for subsequent data analyses to prevent bias.

Reporting for specific materials, systems and methods

We require information from authors about some types of materials, experimental systems and methods used in many studies. Here, indicate whether each material, system or method listed is relevant to your study. If you are not sure if a list item applies to your research, read the appropriate section before selecting a response.

Materials & experimental systems

n/a	Involved in the study	
☐	☒	Antibodies
☐	☒	Eukaryotic cell lines
☒	☐	Palaeontology and archaeology
☐	☒	Animals and other organisms
☒	☐	Clinical data
☒	☐	Dual use research of concern

Methods

n/a	Involved in the study	
☒	☐	ChIP-seq
☒	☐	Flow cytometry
☒	☐	MRI-based neuroimaging

Antibodies

Antibodies used	Primary antibodies used included SARS nucleocapsid protein (1:800, Sino Biologicals, 40143-MM05), MX1 (1:100, Proteintech, 13750-1-AP), prosurfactant protein C (1:200, EMD MIllipore, AB3786), Iba1 (1:100, Abcam, ab5076), RAGE (1:400, Abcam, ab216329), E-cadherin (ECAD) (1:50, Abcam, ab219332), anti-digoxigenin antibody (1:100, #11093274910, Roche Molecular Biochemicals, Indianapolis, IN), and primary rabbit anti-N Wuhan-1 antibody (1:2,000, #U739BGB150, Genscript). Secondary antibodies include Alexa Fluor 488 (1:200 A-21206, 1:2000 A-11008), Alexa Fluor 647 (1:200, A-31571 and A-21447) (Thermofisher, Waltham, MA), 1µg/ml mAb (MTH29-biotin), 1:4000 HRP-conjugated goat anti-hamster IgG (Cat No. 6060-05, Southern Biotech, Birmingham, AL), 1:4000 rabbit anti-hamster IgA (Cat. No. sab 3001a, Brookwood Biomedical, Jemison, AL), 1:4000 HRP-conjugated goat anti-rabbit IgG (4030-05, Southern Biotech, Birmingham, AL), were used based on manufacturers' recommendations.
Validation	The applications of these antibodies were validated by the vendors on the manufacturers' websites or data provided in the manuscript.

Eukaryotic cell lines

Policy information about cell lines and Sex and Gender in Research

Cell line source(s)	Vero E6 cell line (CRL-1586) and Calu-3 cell line (HTB-55) were purchased from American Type Cell Collection (ATCC). H1299-hACE2 is a human lung carcinoma cell line stably expressing human ACE2. It was made by S. Liu in our group from NCI-1299 human lung carcinoma cell line (CRL-5803) purchased from ATCC. Lenti-X 293T cell line was purchased from Takarabio (Cat No. 632180). 293T-hACE2 and 293T-mACE2 cell lines were made from lenti-X 293T cells and published elsewhere (Liu S, et al. Cell Reports. 2022).
Authentication	Vendors routinely validate these cell lines using PCR assays with species-specific primers.
Mycoplasma contamination	No Mycoplasma contamination as verified by PCR
Commonly misidentified lines (See ICLAC register)	None

Animals and other research organisms

Policy information about studies involving animals; ARRIVE guidelines recommended for reporting animal research, and Sex and Gender in Research

Laboratory animals	Adult male outbred Syrian hamsters were previously purchased from Envigo and held at FDA vivarium. All experiments were performed within the biosafety level 3 (BSL-3) suite on the White Oak campus of the U.S. Food and Drug Administration. The animals were implanted subcutaneously with IPTT-300 transponders (BMDS), randomized, and housed 2 per cage in sealed, individually ventilated rat cages (Allentown). Hamsters were fed irradiated 5P76 (Lab Diet) ad lib, housed on autoclaved aspen chip bedding with reverse osmosis-treated water provided in bottles, and all animals were acclimatized at the BSL3 facility for 4-6 days or more prior to the experiments.<br><br>Adult male Balb/c mice were purchased from Jackson, vaccinated with MVA-S, then transferred to BSL-3 for the boost with LAV candidates. All experiments with live-attenuated SARS-CoV-2 were performed within the biosafety level 3 (BSL-3) suite on the White Oak campus of the U.S. Food and Drug Administration while initial vaccination with MVA-S occurred in BSL2 facilities at the FDA vivarium. The animals were housed in groups of up to 5 in sealed, individually ventilated cages. Cages were maintained under controlled light (12:12 Light/Dark cycle), temperature (72°F ± 1°F), and humidity (30-70%) conditions. Mice were fed and watered ad libitum with environmental enrichment, and all animals were acclimatized at the BSL3 facility for 4-6 days or more prior to the experiments.
Wild animals	No wild animals were used in this study.
Reporting on sex	Male outbred Syrian hamsters and Balb/c mice were used in this study.
Field-collected samples	No field collected samples were used.
Ethics oversight	The study protocol details for Syrian hamsters (ASP2020-06) and mice (ASP 2020-30 and ASP 2008-02) were approved by the White Oak Consolidated Animal Care and Use Committee and carried out in accordance with the PHS Policy on Human Care & Use of Laboratory Animals.

Note that full information on the approval of the study protocol must also be provided in the manuscript.
