# Supplementary material for: Intranasal or airborne transmission-mediated delivery of an attenuated SARS-CoV-2 protects Syrian hamsters against new variants
Source: Nat Commun. 2023 Jun 9;14:3393. doi: 10.1038/s41467-023-39090-4 (PMC10250859; doi:10.1038/s41467-023-39090-4)
Supplement: Supplementary file 4 — Description of Additional Supplementary Files [file 41467_2023_39090_MOESM4_ESM.pdf]

Description of Additional Supplementary Files:

Supplementary Movie 1: Illustration of airborne transmission. Dry ice vapor moves across the metal divider, left to right. Air flows into the middle lower back part of the cage and exhausts at the upper lid to the right of center through a black filter. This video was taken as the cage air is being completely replaced 40-60 times per hour (normal ventilated cage flow rate).
